# Supplementary material for: Evolution of the Morphology and Magnetic Properties of Flaky FeSiAl/MFe2O4 (M = Mn, Co, Ni, Cu, Zn) Composites
Source: Nanomaterials (Basel). 2023 Feb 13;13(4):712. doi: 10.3390/nano13040712 (PMC9966722; doi:10.3390/nano13040712)
Supplement: Supplementary file 1 [file nanomaterials-13-00712-s001.zip › nanomaterials-2163495-supplementary.pdf]

Evolution of the Morphology and Magnetic Properties of Flaky FeSiAl/MFe_2O_4 (M = Mn, Co, Ni, Cu, Zn) Composites

Chuannan Ge [1], Chenglong Lei [1,2,*], Bo Wang [1], Yakun Wang [1], Zhouhao Peng [1], Zhitong Wang [1] and Yunjun Guo [1]

[1] School of Physics and Information Engineering, Jiangsu Second Normal University, Nanjing 210013, China
[2] Haian Institute of High-Tech Research, Nanjing University, Nanjing 210093, China
* Correspondence: leichenglong@nju.edu.cn

Figure S1

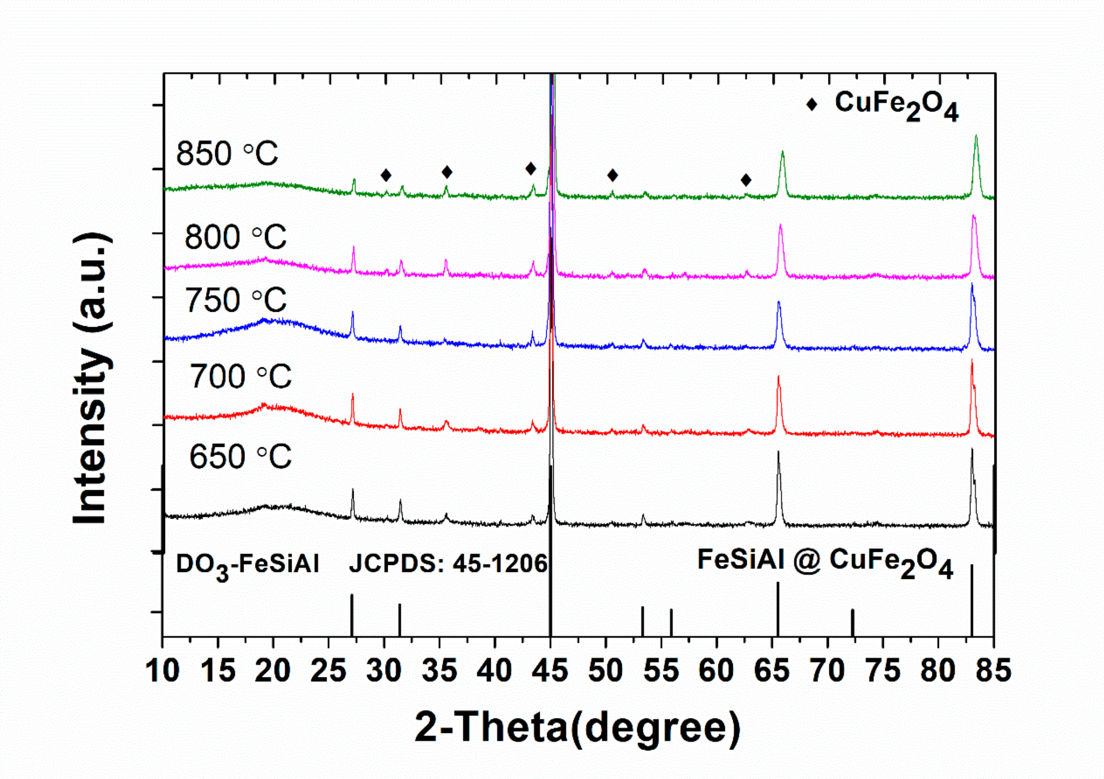

Fig. S1. XRD pattern of flaky FeSiAl composites coated with $CuFe_2O_4$ ferrites annealing at 650, 700, 750, 800 and 850°C.
